# College and Hospital Notes

**Published:** 1907-05

**Authors:** 


### COLLEGE AND HOSPITAL NOTES.

The medical building of McGill University, Montreal, was practically destroyed by fire April 16, 1907. The museum, one of the best on the continent, was also destroyed, but the valuable medical library was saved. This was the second disastrous fire within two weeks, the former resulting in the loss of the McDonald engineering building. The destruction of these buildings is a distinct loss to medical science and to the Dominion of Canada.

Dr. T. O. Maxwell, formerly superintendent of the Southwestern Insane Asylum at San Antonio, Texas, and for sixteen years first assistant physician to the State Insane Asylum at Austin, Texas, has purchased an interest in the Texas Sanitarium for Tuberculosis, Llano, Texas, and has been elected medical director of that institution, taking active charge of same.

